# The Role of Nitrogen-Efficient Cultivars in Sustainable Agriculture

**DOI:** 10.1100/tsw.2001.264

**Published:** 2001-11-06

**Authors:** Franz Weisler, Torsten Behrens, Walter J. Horst

**Affiliations:** Institute of Plant Nutrition, University of Hannover, Germany

**Keywords:** Brassica napus, canola, cultivar, efficiency, genotype, leaching, maize, nitrate, nitrogen, oilseed rape, photosynthesis, uptake, utilization, yield, Zea mays

## Abstract

To improve nitrogen (N) efficiency in agriculture, integrated N management strategies that take into consideration improved fertilizer, soil, and crop management practices are necessary. This paper reports results of field experiments in which maize (Zea mays L.) and oilseed rape (Brassica napus L.) cultivars were compared with respect to their agronomic N efficiency (yield at a given N supply), N uptake efficiency (N accumulation at a given N supply), and N utilization efficiency (dry matter yield per unit N taken up by the plant). Under conditions of high N supply, significant differences among maize cultivars were found in shoot N uptake, soil nitrate depletion during the growing season, and the related losses of nitrate through leaching after the growing season. Experiments under conditions of reduced N supply indicated a considerable genotypic variation in reproductive yield formation of both maize and oilseed rape. High agronomic efficiency was achieved by a combination of high uptake and utilization efficiency (maize), or exclusively by high uptake efficiency (rape). N-efficient cultivars of both crops were characterized by maintenance of a relatively high N-uptake activity during the reproductive growth phase. In rape this trait was linked with leaf area and photosynthetic activity of leaves. We conclude that growing of N-efficient cultivars may serve as an important element of integrated nutrient management strategies in both low- and high-input agriculture.

## INTRODUCTION

Nitrogen (N) is the most limiting nutrient for crop production in many of the world's agricultural areas. To meet the food needs of a growing world population, global use of N fertilizers increased largely during the past 4 decades[1]. However, the efficiency of fertilizer N is frequently small, with often less than 50% of the applied N taken up by the crop[2,3]. This may cause severe yield limitations where there is a lack of N fertilizers and may increase the risk of environmental pollution of both air and water, particularly where high N fertilizer doses are applied to achieve maximum yields. To improve N efficiency in agriculture, integrated N management strategies that take into consideration improved fertilizer, soil, and crop management practices are necessary[3,4,5] (Table 1). Among these, breeding and growing of N-efficient cultivars may play an important role in both low- (improving crop productivity) and high-input (reduction of environmental pollution) agriculture[6]. Improving N efficiency is of special relevance with those crops being characterized by low recoveries of soil and fertilizer N in harvested organs, i.e., large N balance surpluses. In German agriculture these are silage maize and oilseed rape. In maize, N balance surpluses have been attributed to long-term applications of high rates of animal manure and slurry, resulting in both an excessive direct N supply and an increase of the N mineralization potential of the soil. In oilseed rape production, N balance surpluses have mainly been attributed to low nitrogen harvest indices (NHI), i.e., high total N uptake but low N export with harvested seeds[7,8]. This paper reports results of our field experiments in which maize and oilseed rape cultivars were compared with respect to their agronomic N efficiency (yield at a given N supply), N uptake efficiency (N accumulation at a given N supply), and N utilization efficiency (reproductive yield per unit N taken up by the plant).

## MATERIALS AND METHODS

Three field experiments (Exp. 1, 2, and 3) were conducted with commercial maize (*Zea mays* L.) and oilseed rape (*Brassica napus* L.) cultivars. Fertilizers were applied in the form of urea-ammonium nitrate solution (Exp. 1) or calcium ammonium nitrate (Exp. 2, 3). N concentration in the plant dry matter was measured with a CNS analyzer.

In Exp. 1, ten maize cultivars from the maturity groups "early," "medium early," and "medium late" (silage maize production) were grown for two seasons on a Gleyic Luvisol in Stuttgart, southern Germany, at high soil N supply (295 kg $NO_3$-N $ha^{-1}$ in 0 to 120 cm soil depth at the beginning of both seasons). Nitrate leaching at 120 cm soil depth was calculated during the entire experimental period (May 1987 to April 1989) on the basis of soil nitrate concentrations in suction cup water and water drainage at 120 cm soil depth (sampling of suction cup water in 2-week intervals, recording of soil water matric potentials with tensiometers in weekly intervals). Additionally, soil nitrate-N contents in soil samples were periodically monitored using $CaCl_2$ extraction. A more detailed description of methods is given by Wiesler and Horst[9].

In Exp. 2, three selected maize cultivars from the maturity group "medium early" (DK 240, Green and Lixis; kernel maize production) were grown for two seasons on a Luvisol in the Rhine valley, 30 km south of Freiburg, southern Germany. The three

**TABLE 1**
**Elements of Integrated Nutrient Management Strategies that May Result in Improved N Efficiency in Plant Production**

- Crop and crop rotation:
  → Increased uptake and utilization of soil and fertilizer N by cultivation of N-efficient crops, reduction of fallow frequency and rotation of shallow/deep rooting crops.
- Cultivar:
  → Increased uptake and utilization of soil and fertilizer N by cultivation of N-efficient cultivars.
- Irrigation and crop protection:
  → Increased uptake of soil and fertilizer N by well-grown crops.
- Accurate prediction of fertilizer N demand (e.g., soil and plant tests, sensor controlled fertilization ("precision farming"), modeling soil N supply):
  → Increased uptake of soil and fertilizer N by considering available soil mineral N at the beginning of the growing season and N mineralization during the growing season.
- Form of N fertilizer (e.g., mineral fertilizers vs. organic manure, urea vs. ammonium vs. nitrate fertilizers, use of urease and nitrification inhibitors):
  → Avoidance of N losses caused by specific N forms/N transformations in the soil, increased physiological efficiency of N by considering plant species specific preferences of certain forms of N ($NH_4^+$ vs. $NO_3^-$).
- Timing of N application:
  → Reduction of N losses ($NO_3^-$, $N_2$) at the beginning of the growing season, increased physiological efficiency by specific growth stimulation of harvestable organs.
- Technique of N application (e.g., surface vs. incorporation, broadcast vs. banded):
  → Reduction of N losses ($NH_3$), improved spatial availability of N, reduction of N immobilization.
- Cover crops/intercropping:
  → Uptake of soil N and mineralized plant residue N during autumn and thereby reducing N losses by leaching and increasing N supply to succeeding crops.
- Management of crop residues:
  → Control of N mineralization during autumn/winter.
- Incorporation of straw:
  → Immobilization of soil mineral N.
- Timing, intensity, and depth of soil cultivation:
  → Control of soil N mineralization.

cultivars were compared at low (N1: 86 and 26 kg N $ha^{-1}$ in 1994 and 1995), medium (N2: 146 and 180 kg N $ha^{-1}$ in 1994 and 1995), and high (N3: 206 and 224 kg N $ha^{-1}$ in 1994 and 1995) N supply. N supply includes fertilizer N and the initial mineral N content in 0- to 60-cm soil depth at the beginning of the growing season.

In Exp. 3, two winter oilseed rape cultivars (Apex, Capitol) were grown for two seasons near Göttingen, northern Germany. Similarly to Exp. 2, cultivars were compared at low (N1: 20 and 17 kg N $ha^{-1}$ in 1998 and 1999), medium (N2: 120 kg N $ha^{-1}$), and high (N3: 240 kg N $ha^{-1}$) N supply. Fertilizer N was split applied in early spring (March) and at the beginning of shooting (April). In 2000, the photosynthetic activity of leaves (LI-6400 portable photosynthesis system) and leaf chlorophyll-concentrations (SPAD-502 chlorophyll meter) of the two cultivars were measured at the end of flowering. Gas exchange measurements were carried out under controlled light (photosynthetic active radiation: 1000 μmol quanta $m^{-2}$ $s^{-1}$) and $CO_2$ (incoming $CO_2$ concentration: 360 μmol $mol^{-1}$) conditions. All leaves of one representative plant per plot were considered.

Each experiment was conducted with four replicates, either in the form of a randomized block design (Exp. 1) or in the form of split-plot designs (Exp. 2, 3). Results were subjected to analyses of variance. In Tables 2 through 7, results of the F test (***, **, and * indicate significance at the $p < 0.001$, 0.01, and 0.05 levels) and the Tukey test (LSD, $p < 0.05$) are given. For a comparison of cultivars, data of years have been pooled (Exp. 2, 3) as there were only a few significant year × cultivar interactions.

## RESULTS AND DISCUSSION

### Differences Among Maize Cultivars in Shoot N Uptake and the Related Losses of Nitrate Through Leaching (Exp. 1)

Significant differences ($p < 0.05$) in total shoot N were found among the ten maize cultivars, the ranges being 177 to 223 kg $ha^{-1}$ at the end of the 1987 season and 185 to 226 kg $ha^{-1}$ at the end of the 1988 season. Cultivars with high shoot N uptake were characterized by high stover yields (r = 0.83** in 1987 and 0.72* in 1988) and a high root length at silking, particularly in the 60 to 90 cm subsoil layer (r = 0.88*** in 1987 and 0.69* in 1988). Ear yield was negatively correlated with shoot N uptake (r = –0.46 in 1987 and –0.72* in 1988). In both years, shoot N uptake of the cultivars was significantly positively correlated with soil nitrate depletion during the growing season (r = 0.82** in 1987 and 0.83*** in 1988) and negatively correlated with the residual soil nitrate-N content at final harvest in October (Fig. 1). In both years, nitrate leaching at 120 cm soil depth was negligible during shooting and the reproductive growth phase. Leaching started soon after harvest and continued until June 1988 and April 1989. The calculated amount of nitrate-N leached below 120 cm soil depth varied between 57 and 84 kg N $ha^{-1}$ during the 1987/88 leaching period, and between 47 and 79 kg N $ha^{-1}$ during the 1988/89 leaching period. In agreement with studies comparing fertilization practices[10], nitrate leaching was closely related to residual soil nitrate-N on the plots of the ten cultivars after harvest. We conclude from this study that growing of cultivars capable of utilizing a high N supply, particularly at deeper soil layers, may substantially contribute to a reduction of nitrate losses through leaching.

### Differences Among Maize Cultivars in Yield Formation, N Uptake, and N Utilization Efficiency at Low and High N Supply (Exp. 2)

Growing of cultivars capable of utilizing a high soil N supply is of particular importance under conditions of long-term excessive N supply, especially in areas with intensive animal production. However, in the future, sustainable cropping systems will require a better balancing of N input and output with harvested organs, making it necessary to produce higher yields with less N[4]. Under these conditions, improvements of both N uptake and utilization efficiency are of fundamental importance. We compared three selected maize cultivars for these traits in two seasons.

Experimental conditions clearly influenced growth and yield in the two growing seasons, particularly in the N1 treatment (Table 2). This may be explained by the different initial soil mineral N contents in the 2 years. However, irrespective of year, N application (N2, N3) resulted in significantly increased stover and grain dry weights at maturity compared with those in the N1 treatment. Differences between the N2 and N3 treatments were small. No significant differences between the cultivars existed in stover dry matter at low N supply, whereas Lixis produced a higher vegetative biomass than DK 240 in the N2 and N3 treatments. In clear contrast, DK 240 produced a higher grain dry matter than Green and especially Lixis at each fertilizer N level. Similarly, NHIs were highest for DK 240, lowest for Lixis, and intermediate for Green (not shown). In agronomic terms DK 240 can be defined as N-efficient because it converted the same N input into a higher output of harvested grain than Green and Lixis. Additionally, relative stover and grain dry matter production in the N1 treatment (N3 = 100) was slightly higher for DK 240 (76 and 70%) than for Green (71 and 68%) and Lixis (70 and 67%), indicating that the efficient cultivar responded less sensitively to a reduced N supply.

To explain differences among the cultivars in their agronomic N efficiency, shoot N concentrations, shoot N uptake, and N utilization were measured/calculated. Increasing the N supply increased shoot N concentrations at silking and both straw and grain N concentrations at maturity (Table 3). Differences between years were not consistent over N treatments. Differences among cultivars were relatively small at silking but significant at maturity in both stover and grains. Interestingly, N concentrations in the vegetative plant parts of the N-efficient cultivar DK 240 declined in a less pronounced manner than in Green and Lixis during the reproductive growth phase, resulting in the highest stover N concentrations at maturity in all N treatments. By contrast, grain N concentrations were significantly higher in Green and Lixis. Higher stover N concentrations were in line with higher SPAD meter values (data not shown). Similar results were obtained by Ma and Dwyer[11] who showed that grain yields of a stay-green

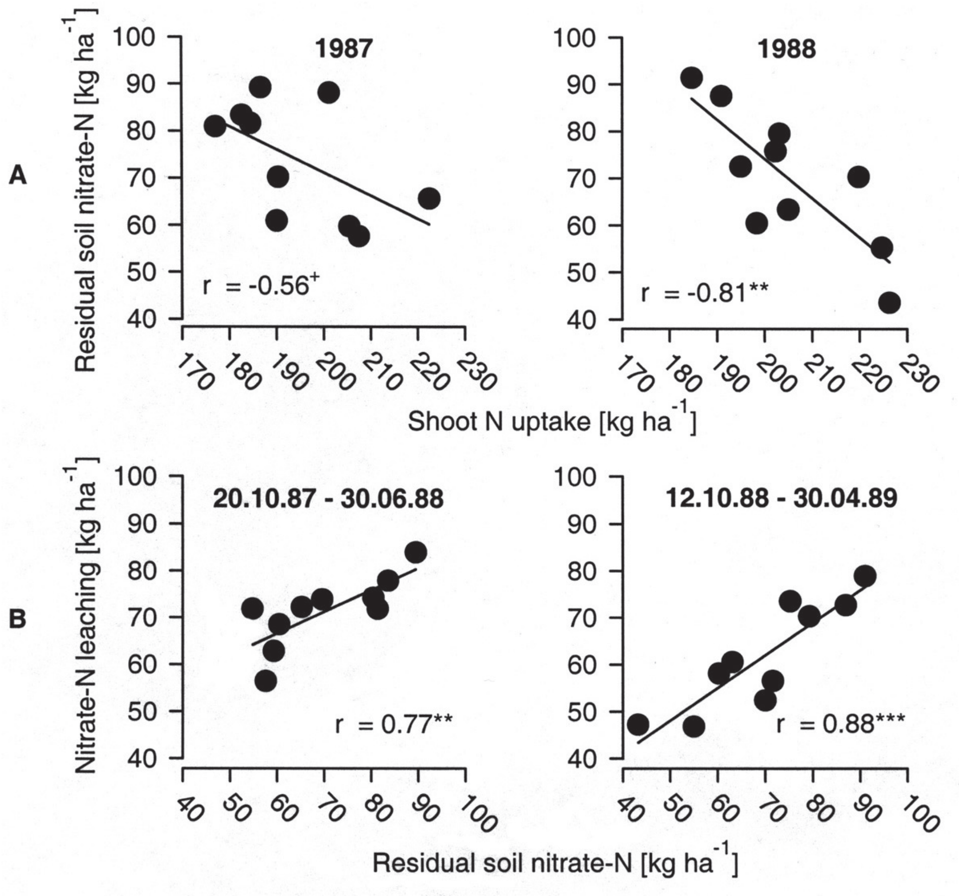

**FIGURE 1.** Relationships between shoot N uptake and residual nitrate-N contents on the plots of ten maize cultivars (A) and relationships between residual nitrate-N contents and nitrate-N leaching during the 1987/88 and 1988/89 leaching periods (B). ***, **, and + indicate significance at the $p < 0.001$, 0.01, and 0.1 level. Soil nitrate-N and leaching data from Wiesler and Horst[9].

**TABLE 2**
**Straw and Grain Dry Matter of Maize as Influenced by Year (Y), Cultivar (C), and N Supply**

| | Year | | | Cultivar | | | | F Test | | |
|---|---|---|---|---|---|---|---|---|---|---|
| N Supply | 1994 | 1995 | LSD | DK 240 | Green | Lixis | LSD | Y | C | Y x C |
| **Straw dry matter [t ha⁻¹]** | | | | | | | | | | |
| N1 | 7.4 a | 5.2 b | 0.4 | 6.3 a | 6.0 a | 6.4 a | 0.6 | *** | n.s. | n.s. |
| N2 | 8.7 a | 8.5 a | 0.8 | 7.9 b | 8.8 ab | 9.2 a | 1.2 | n.s. | * | n.s. |
| N3 | 8.9 a | 8.4 a | 0.7 | 8.3 a | 8.5 a | 9.2 a | 1.0 | n.s. | n.s. | n.s. |
| **Grain dry matter [t ha⁻¹]** | | | | | | | | | | |
| N1 | 8.5 a | 4.7 b | 0.5 | 7.3 a | 6.6 a | 5.8 b | 0.7 | *** | *** | n.s. |
| N2 | 10.0 a | 8.2 b | 0.6 | 10.2 a | 8.9 b | 8.3 b | 0.9 | *** | *** | n.s. |
| N3 | 10.8 a | 8.3 b | 0.7 | 10.4 a | 9.7 b | 8.6 c | 0.7 | *** | *** | * |

*Note*: Means within N levels with the same letter are not significantly different

**TABLE 3**
**N Concentration in Shoot Dry Matter at Silking, and in Straw and Grain Dry Matter at Maturity of Maize as Influenced by Year (Y), Cultivar (C), and N Supply**

| | Year | | | Cultivar | | | | F Test | | |
|---|---|---|---|---|---|---|---|---|---|---|
| N Supply | 1994 | 1995 | LSD | DK 240 | Green | Lixis | LSD | Y | C | Y x C |
| **Shoot N concentration at silking [mg g$^{-1}$ dry matter]** | | | | | | | | | | |
| N1 | 16.9 a | 17.6 a | 1.2 | 16.9 a | 17.6 a | 17.2 a | 1.8 | n.s. | n.s. | n.s. |
| N2 | 17.4 b | 21.0 a | 1.1 | 18.2 b | 18.9 ab | 20.4 a | 1.6 | *** | ** | n.s. |
| N3 | 23.0 a | 21.4 b | 0.9 | 22.8 a | 22.0 a | 21.7 a | 1.4 | ** | n.s. | n.s. |
| **Stover N concentration at maturity [mg g$^{-1}$ dry matter]** | | | | | | | | | | |
| N1 | 6.9 a | 5.4 b | 0.5 | 7.0 a | 5.7 b | 5.7 b | 0.8 | *** | ** | * |
| N2 | 7.4 a | 7.5 a | 0.6 | 7.9 a | 6.9 b | 7.5 ab | 0.9 | n.s. | * | * |
| N3 | 7.4 b | 8.2 a | 0.6 | 8.7 a | 7.5 b | 7.2 b | 0.9 | * | ** | n.s. |
| **Grain N concentration at maturity [mg g$^{-1}$ dry matter]** | | | | | | | | | | |
| N1 | 11.6 a | 10.0 b | 0.6 | 9.9 b | 11.5 a | 11.1 a | 0.9 | *** | ** | n.s. |
| N2 | 12.9 b | 13.6 a | 0.5 | 12.1 b | 13.8 a | 13.7 a | 0.8 | * | *** | * |
| N3 | 13.7 b | 14.5 a | 0.7 | 13.1 b | 14.5 a | 14.7 a | 1.1 | * | ** | n.s. |

hybrid were higher than those of an early senescing hybrid at both low and high N supply.

Delayed senescence as found for DK 240 might have been caused by higher N uptake during the reproductive growth phase and/or delayed remobilization of N from vegetative to reproductive organs. At silking, shoot N uptake tended to be lower in DK 240 than in Green and Lixis (not shown). By contrast, at maturity shoot N uptake tended to be largest in DK 240, particularly at N1 and when compared with Lixis (Table 4). This may be explained by a higher N uptake of DK 240 during the reproductive growth phase. Averaged over N treatments, cultivars accumulated 45 (DK 240), 19 (Green), and 12 (Lixis) kg N ha$^{-1}$ after silking (no significant cultivar × N interaction). This accounts for 23, 8, and 4% of total shoot N at maturity. NHIs indicate that grains contained 62 to 69% of total shoot N with only small and inconsistent differences among cultivars. Irrespective of N treatment (no N × cultivar interaction), DK 240 produced a significantly higher grain dry matter per kilogram shoot N than Green and Lixis, indicating that both uptake and utilization efficiency contributed to the higher agronomic efficiency of this cultivar. These results are in contrast to the classical studies of Moll et al.[12] who found that agronomic N efficiency of maize hybrids was due largely to N utilization efficiency at low N supply and to uptake efficiency at high N supply.

## Differences Among Oilseed Rape Cultivars in Yield Formation, N Uptake, and N Utilization at Low and High N Supply (Exp. 3)

Oilseed rape has been characterized as a crop with high N uptake until flowering, low N uptake during the reproductive growth phase, and incomplete N retranslocation from vegetative plant parts to the seeds, resulting in comparatively low NHIs and large N balance surpluses[7,8]. In contrast to maize, the examination of genotypical differences in N efficiency of rape has received little attention in the past[13]. To compare cultivars with respect to seed yield formation, N uptake, and N utilization, two cultivars were grown in a 2-year experiment at low, medium, and high N supply.

Irrespective of N supply, growing conditions in 1999 clearly favored yield formation (Table 5), N uptake, and N utilization (Table 7) compared to the 1998 growing season. In both years, seed yield, straw and seed N concentration (Table 6), and shoot N uptake increased up to the highest N rate. By contrast, N utilization decreased with increasing N supply.

A comparison of cultivars revealed similar straw dry matters at maturity, but with respect to seed dry matter, Apex out-

**TABLE 4**
**Shoot N Uptake at Maturity, N Uptake between Silking and Maturity, NHI and N Utilization of Maize as Influenced by Year (Y), Cultivar (C), and N Supply**

| | Year | | | Cultivar | | | | F Test | | |
|---|---|---|---|---|---|---|---|---|---|---|
| N Supply | 1994 | 1995 | LSD | DK 240 | Green | Lixis | LSD | Y | C | Y x C |
| **Shoot N content at maturity [kg ha$^{-1}$]** | | | | | | | | | | |
| N1 | 150 a | 74 b | 9 | 120 a | 114 ab | 103 b | 13 | *** | * | n.s. |
| N2 | 194 a | 174 b | 14 | 186 a | 184 a | 182 a | 21 | * | n.s. | * |
| N3 | 213 a | 189 b | 11 | 207 a | 204 a | 193 a | 17 | *** | n.s. | n.s. |
| **N uptake between silking and maturity [kg ha$^{-1}$]** | | | | | | | | | | |
| N1 | 27 a | –4 b | 19 | 27 a | 6 a | 2 a | 28 | ** | n.s. | n.s. |
| N2 | 57 a | 9 b | 12 | 65 a | 27 b | 20 b | 35 | ** | ** | n.s. |
| N3 | 27 a | 29 a | 23 | 44 a | 25 a | 15 a | 35 | n.s. | n.s. | n.s. |
| **N harvest index** | | | | | | | | | | |
| N1 | 0.66 a | 0.62 b | 0.02 | 0.62 b | 0.63 b | 0.68 a | 0.03 | ** | *** | * |
| N2 | 0.67 a | 0.64 a | 0.04 | 0.66 a | 0.67 a | 0.62 a | 0.06 | n.s. | n.s. | n.s. |
| N3 | 0.69 a | 0.64 b | 0.04 | 0.65 a | 0.69 a | 0.66 a | 0.05 | ** | n.s. | n.s. |
| **N utilization [kg grain dry matter (kg total plant N)$^{-1}$]** | | | | | | | | | | |
| N1 | 57 b | 62 a | 4 | 63 a | 60 a | 57 a | 7 | * | n.s. | n.s. |
| N2 | 52 a | 47 b | 3 | 55 a | 49 b | 45 b | 5 | ** | ** | n.s. |
| N3 | 51 a | 44 b | 3 | 50 a | 47 ab | 45 b | 5 | *** | * | n.s. |

**TABLE 5**
**Straw and Seed Dry Matter of Oilseed Rape as Influenced by Year (Y), Cultivar (C), and N Supply**

| | Year | | | Cultivar | | | F Test | | |
|---|---|---|---|---|---|---|---|---|---|
| N Supply | 1998 | 1999 | LSD | Apex | Capitol | LSD | Y | C | Y x C |
| **Straw dry matter [t ha$^{-1}$]** | | | | | | | | | |
| N1 | 3.61 a | 4.07 a | 0.70 | 3.90 a | 3.78 a | 0.70 | n.s. | n.s. | n.s. |
| N2 | 4.28 b | 6.05 a | 1.00 | 5.00 a | 5.46 a | 1.00 | ** | n.s. | n.s. |
| N3 | 5.27 a | 5.42 a | 0.61 | 5.62 a | 5.09 a | 0.61 | n.s. | n.s. | n.s. |
| **Seed dry matter [t ha$^{-1}$]** | | | | | | | | | |
| N1 | 2.14 b | 2.98 a | 0.49 | 2.96 a | 2.15 b | 0.49 | ** | ** | n.s. |
| N2 | 2.38 b | 4.20 a | 0.44 | 3.55 a | 3.00 b | 0.44 | *** | * | n.s. |
| N3 | 2.78 b | 4.94 a | 0.60 | 4.13 a | 3.59 a | 0.60 | *** | n.s. | n.s. |

**TABLE 6**
**N Concentration in Shoot Dry Matter at Flowering, and in Straw and Seed Dry Matter at Maturity of Rape as Influenced by Year (Y), Cultivar (C), and N Supply**

| | Year | | | Cultivar | | | F Test | | |
|---|---|---|---|---|---|---|---|---|---|
| N Supply | 1998 | 1999 | LSD | Apex | Capitol | LSD | Y | C | Y x C |
| **Shoot N concentration at the beginning of flowering [mg g$^{-1}$ dry matter]** | | | | | | | | | |
| N1 | 19.4 a | 14.6 b | 1.6 | 17.2 a | 16.8 a | 1.6 | *** | n.s. | n.s. |
| N2 | 27.2 a | 20.8 b | 1.5 | 26.0 a | 22.0 b | 1.5 | *** | *** | n.s. |
| N3 | 34.9 a | 29.4 b | 2.3 | 34.7 a | 29.7 b | 2.3 | ** | ** | n.s. |
| **Straw N concentration at maturity [mg g$^{-1}$ dry matter]** | | | | | | | | | |
| N1 | 5.2 a | 3.0 b | 0.5 | 4.3 a | 3.8 a | 0.5 | *** | n.s. | * |
| N2 | 5.4 a | 4.1 a | 1.3 | 5.9 a | 3.6 b | 1.3 | n.s. | ** | n.s. |
| N3 | 8.0 a | 6.4 b | 1.3 | 8.1 a | 6.3 b | 1.3 | * | * | n.s. |
| **Seed N concentration at maturity [mg g$^{-1}$ dry matter]** | | | | | | | | | |
| N1 | 29.5 a | 23.8 b | 1.1 | 26.2 a | 27.2 a | 1.1 | *** | n.s. | * |
| N2 | 29.6 a | 25.5 b | 1.2 | 27.4 a | 27.7 a | 1.2 | *** | n.s. | n.s. |
| N3 | 34.6 a | 31.9 b | 2.7 | 33.1 a | 33.4 a | 2.7 | n.s. | n.s. | n.s. |

**TABLE 7**
**Shoot N Uptake at Maturity, N Uptake between Beginning of Flowering and Maturity, NHI, and N Utilization of Rape as Influenced by Year (Y), Cultivar (C), and N Supply**

| | Year | | | Cultivar | | | F Test | | |
|---|---|---|---|---|---|---|---|---|---|
| N Supply | 1998 | 1999 | LSD | Apex | Capitol | LSD | Y | C | Y x C |
| **Shoot N content at maturity [kg ha$^{-1}$]** | | | | | | | | | |
| N1 | 81 a | 83 a | 13 | 93 a | 71 b | 13 | n.s. | ** | n.s. |
| N2 | 92 b | 132 a | 13 | 124 a | 100 b | 13 | *** | ** | n.s. |
| N3 | 138 b | 192 a | 25 | 181 a | 150 b | 25 | ** | * | n.s. |
| **N uptake between beginning of flowering and maturity [kg ha$^{-1}$]** | | | | | | | | | |
| N1 | 17 a | 12 a | 16 | 35 a | –6 b | 16 | n.s. | *** | n.s. |
| N2 | –2 a | –5 a | 20 | 4 a | –11 a | 20 | n.s. | n.s. | n.s. |
| N3 | 11 a | 4 a | 35 | 11 a | 4 a | 35 | n.s. | n.s. | n.s. |
| **N harvest index** | | | | | | | | | |
| N1 | 0.77 b | 0.86 a | 0.03 | 0.83 a | 0.80 a | 0.03 | *** | n.s. | ** |
| N2 | 0.75 a | 0.81 a | 0.08 | 0.77 a | 0.80 a | 0.08 | n.s. | n.s. | n.s. |
| N3 | 0.69 b | 0.82 a | 0.07 | 0.74 a | 0.77 a | 0.07 | ** | n.s. | n.s. |
| **N utilization [kg seed dry matter (kg total plant N)$^{-1}$]** | | | | | | | | | |
| N1 | 26 b | 36 a | 1.7 | 32 a | 30 a | 1.7 | *** | n.s. | ** |
| N2 | 26 b | 32 a | 2.6 | 28 a | 30 a | 2.6 | * | n.s. | n.s. |
| N3 | 20 b | 26 a | 3.5 | 23 a | 23 a | 3.2 | ** | n.s. | n.s. |

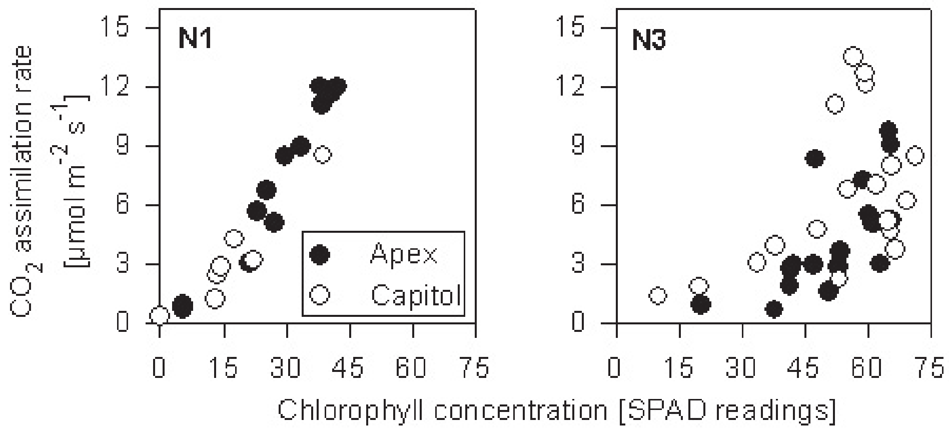

**FIGURE 2.** Chlorophyll concentration and photosynthetic activity of individual leaves of two oilseed rape cultivars at low (N1) and high (N3) N supply at the end of flowering.

yielded Capitol significantly at low and medium N supply. Thus, HIs were higher for Apex than for Capitol, particularly in the N1 (0.44 vs. 0.36) and N2 (0.42 vs. 0.36) treatments.

In agreement with Exp. 2, straw N concentrations were higher in the efficient cultivar Apex compared with those in Capitol (Table 6), again indicating that senescence in the efficient cultivar was delayed. Only small differences among the cultivars were found in seed N concentrations.

Shoot N uptake as measured at the beginning of flowering was lower in Apex at low N supply but higher in Capitol at high N supply (not shown). However, at maturity, Apex showed the highest N uptake, irrespective of N supply (Table 7). Calculated N uptake between beginning of flowering and maturity clearly indicates a higher uptake activity of Apex during the reproductive growth phase. Negative uptake of Capitol indicates that N loss by leaf drop exceeded N uptake during the reproductive growth phase. For various environmental conditions, N losses with dropping leaves of 10 to 30 kg N $ha^{-1}$ have been reported[14,15]. NHIs varied between 0.74 and 0.83 and were thus higher than those reported in the literature[7,14]. They would be lower, however, when N losses with dropping leaves would be considered. Differences among the cultivars were small and not significant. N utilization efficiency hardly contributed to the differences in agronomic efficiency of the two cultivars.

Both the experiments with maize and oilseed rape indicate that yield formation is closely related to N uptake efficiency during the reproductive growth phase. Since uptake efficiency depends on assimilate supply for root growth and activity, leaf area indices and photosynthetic activity of leaves of Apex and Capitol were measured during the reproductive growth phase at low and high N supply. Leaf area indices of Apex were regularly higher than those of Capitol (not shown). At low N supply, Apex had more leaves per plant than Capitol with higher chlorophyll values (26 vs. 17) at the end of flowering (Fig. 2). Since leaf chlorophyll concentration and $CO_2$ assimilation rate were closely correlated, mean $CO_2$ assimilation rate of Apex was substantially higher than that of Capitol (6.6 vs. 3.2 µmol $m^{-2}$ $s^{-1}$). In clear contrast, at high N supply, no differences among the two cultivars existed in chlorophyll values (52), and mean $CO_2$ assimilation rate of Apex was even lower than that of Capitol (4.3 vs. 6.5 µmol $m^{-2}$ $s^{-1}$).

## CONCLUSIONS

Examples presented in this paper show that growing of N-efficient cultivars may serve as an important element of integrated nutrient management strategies in both low- and high-input agriculture. Particularly under conditions of excess N supply, growing of cultivars selected for high N-uptake capacity of the shoots combined with an efficient N-acquisition system can help to reduce N losses through leaching. However, future sustainable cropping systems require a better balancing of N inputs and outputs with harvested organs, making it necessary to produce higher yields with less N. Our experiments with maize and oilseed rape indicate a considerable genotypic variation in reproductive yield formation under those conditions. Interestingly, N-efficient cultivars of both crops were characterized by maintenance of a relatively high N-uptake activity during the reproductive growth phase. Prolonged N uptake contributes to a better use of N released from the organic N pool of the soil late in the growing season, and is thus also of benefit from an environmental point of view. Our data suggest that maintenance of green leaf area and photosynthetic activity of leaves are characteristics contributing to prolonged N uptake activity during the reproductive growth phase of N-efficient cultivars. Better knowledge of morphological and physiological plant traits controlling N efficiency is essential for both selection of cultivars and well-directed breeding strategies to improve N uptake and utilization efficiency of crops.

## ACKNOWLEDGEMENTS

The authors wish to thank the Deutsche Forschungsgemeinschaft (DFG) for long-term financial support of this work.

| | | |
|---|---|---|
| Received: | July | 19, 2001 |
| Revised: | September | 29, 2001 |
| Accepted: | October | 3, 2001 |
| Published: | November | 6, 2001 |
